# On Amputation

**Published:** 1807-06

**Authors:** 


### *To the Editors of the Medical and Phyſical Journal.*

GENTLEMEN,

THE general question of amputation embraces a large field of discussion; and though seemingly easily arranged and settled by the systematic writer, is found in practice difficult and embarrassing. And after all perhaps that can be said upon the subject, the mind of the enquirer, when called upon to decide in an individual case, will find difficulties which it had not anticipated. For, says a recent and most respectable authority, "each case as it occurs in practice, is attended with circumstances so peculiar, that it will seldom class with our general aphorisms; and frequently, in the discussion of this subject, the first surgeons are left in doubt and perplexity."

There are, however, circumstances in every case, which, if they could be considered in a detached point of view, would incline one, for, or against the operation.

I am led into this train of thinking, from the case of popliteal aneurism, reported from the Royal Naval Hospital, Deal, in your last number. I agree with the Reporter, who merits the thanks of the profession for his communication, that there was no searching for the bleeding vessel or vessels, "in such a mass of corruption." But I cannot think that the moment for amputation was happily chosen.

The poor wretch, however, it seems, suffered but little from the operation—at least, he was too far gone to express in strong terms, his sense of that suffering. He was in fact in deliquium animi—not to be roused even by the "horrid stimulus of the knife."

Though the patient might lose but little blood in the operation, that little, it would appear, was too much for the vessels to be deprived of. The face of the stump might not indeed throw out much blood, but the operation could not be undertaken without the certain prospect of losing some; and the detached limb even, would bear with it a portion, the loss of which alone, might, at that moment, be irreparable to the system;—for there is a point we conceive, in extreme inanition, a single advance beyond which is positive and immediate destruction.

We would not, however, in this case pretend to say, that amputation "accelerated the patient's doom." But it is cruel, nay, it is horrid, so to mutilate a poor sufferer, reduced almost to his last gasp.

We have no means of knowing from the account before us, whether the bleeding was venous or arterial; nor, if the latter, whether it came immediately into the sac from the direct jet of an artery, or had a retrograde and more indirect exit. Whether the injection of the limb after amputation, would have ascertained this fact, we cannot say; nor is it perhaps material to the present question. Though it might have been satisfactory we think, to have explored the channel through which life ebbed.

We only mean to inculcate now, that we would not undertake an operation of this magnitude, on a patient already sinking under a death-like fainting—not from the impression of fear, or the previous shock of battle, but from an exhaustion the most direct and alarming. We would first think of means to stay for a while the vital torrent. We would for instance, in the case before us, have dilated the cyst to its extent; and would then, after evacuating it of its accumulated filth, have crammed it with graduated compresses of sponge; and with the help of a bandage, would have kept up a certain resistance to the bleeding point or surface.

We are aware of the danger of strongly girding a limb, so newly and largely excited as it were, the blood being thus thrown into numerous and comparatively straitened channels; but we would, according to the principles laid down by John Bell, endeavour to obviate this effect, by an equable support to the very extremities of the toes, or we would keep up a local pressure by the hands of renewed assistants.

The limb, no more than the life of the patient, might not have been saved by such procrastination; but it seems to us, as if it would have been the most rational practice; for if the hæmorrhage could have been restrained even for a while, the patient probably might have recruited a little, and the amputation then have been undertaken with a better prospect of success.

But we must not, in thus expatiating, forget the difficulty of the question we first set out with.—We must rather recur to it; and say, in candor to the Gentleman who has given us this history, "that there are cases in which the most experienced surgeon will have difficulty to determine, whether amputation will save the patient or accelerate his doom. These are cases of fractures, aneurism, and gangrene."

### On a demonstration of the Brain.

I have lately had occasion, at least it has so happened, that I have attended the dissection of the brain of a culprit, given to the surgeons for that purpose. The gentleman who undertook the demonstration, is of unquestionable respectability; and yet, he here committed so many inadvertent mistakes, for I cannot otherwise consider them, as to excite in me the most awkward feelings;—awkward, because I did not consider myself in a situation to interfere. There is no occasion to go into the particulars of this demonstration, it might appear invidious to do so, and the general purpose of these observations, for I have no particular aim, may, I hope, be answered without it.

It is a great pity that any one, not every day accustomed to such a task, should go into it, so full of confidence, without any previous thinking we may be sure, or refreshing the memory with a reference to the best authors; or such at least as he thinks proper to consult.

The anatomy of the brain, from its difficult and rare dissection, as well as from the intricacy of its parts, may very soon be forgot, or be but imperfectly remembered. There may not indeed, practically considered, be that inducement for the practitioner, to hold it, in all its foldings, perfectly in his recollection. Because, if we mistake not, its function being understood, there would be no increased difficulty in going on with a case of brainular affection, from the circumstance of knowing little or nothing of its particular formation. But would any surgeon of character wish to have to avail himself of such an apology? and is not every surgeon liable to be called upon, to investigate its morbid anatomy, before intelligent physicians perhaps, or even rivals in practice? And what must be the situation of that man; a man of honest feeling we will suppose, going into a rencounter of this sort with the certain prospect of discomfiture and disgrace?

We well remember the forcible and impressive manner, in which Sir William Blizard, in his Anatomical Course, was accustomed to urge this point on the minds of his pupils; and it is from an impression there received, that we have never lost sight of its importance and just value.

### Infantile Diseases.

Infantile or juvenile diseases are just now committing great havock around us. The croup, the chin-cough, and the measles; as well as distinct pneumonia, are each daily carrying off a portion of innocent and highly regretted little victims. But if, as an admired reporter observes, in your crowded and debilitated community, "the mortar and pestle" are hourly chargable with multiplied murder, we have in the country, an equal and most satisfactory balance against you. For, if timeously resorted to, we can from much observation assert, that numbers of this class of patients, owe their protracted existence to the lancet, the leech, and the purge.

A Practitioner in Derbyshire.

*April* 13, 1807.

